# MicroRNA-345-3p is a potential biomarker and ameliorates rheumatoid arthritis by reducing the release of proinflammatory cytokines

**DOI:** 10.1186/s13018-023-03797-3

**Published:** 2023-06-01

**Authors:** Jun Ma, Wei Zhao, Xue Pei, XinZhi Li, Wei Zhao

**Affiliations:** 1 Department of Orthopedics, Jiu Quan People’s Hospital, No. 22, West Street, Suzhou District, Jiu Quan City, 735000 Gansu Province China; 2 Department of Orthopaedics, Affiliated Renhe Hospital of China Three Gorges University, Yichang City, 443001 Hubei Province China

**Keywords:** MicroRNA-345-3p, Rheumatoid arthritis, Proinflammatory cytokines, Correlation analysis

## Abstract

**Objectives:**

The study was to explore the influence of microRNA (miR)-345-3p on proinflammatory cytokines in patients with rheumatoid arthritis (RA).

**Methods:**

A total of 32 RA patients and 32 healthy patients were enrolled. Proinflammatory factors in patients’ serum were detected by ELISA, and miR-345-3p was detected by RT-qPCR. The correlation between miR-345-3p expression and proinflammatory factors in RA patients was analyzed. The diagnostic value of miR-345-3p and proinflammatory factors in RA patients was analyzed by receiver operating curve diagnosis. The predictive value of miR-345-3p levels and proinflammatory factors in RA patients was analyzed by multivariate Cox regression. HFLS-RA and HFLS cells were cultured, in which miR-345-3p and proinflammatory cytokines were detected by RT-qPCR. Cell proliferation and apoptosis were determined by CCK-8 and flow cytometry, respectively.

**Results:**

MiR-345-3p was lowly expressed in the serum of RA patients. MiR-345-3p and proinflammatory factors were of diagnostic and predictive values in RA. Elevated miR-345-3p restrained the production of proinflammatory factors of HFLS-RA cells, improved cell proliferation, and reduced apoptosis.

**Conclusion:**

MiR-345-3p is a potential biomarker and ameliorates RA by reducing the release of proinflammatory cytokines.

## Highlights

1. miR-345-3p was reduced in the serum of RA patients;
2. Serum miR-345-3p in RA patients was negatively correlated with proinflammatory factors;
3. MiR-345-3p and proinflammatory factors were of diagnostic value in RA;
4. Elevated miR-345-3p suppressed the release of proinflammatory cytokines in HFLS-RA cells;
5. MiR-345-3p ameliorated RA.

## Introduction

Rheumatoid arthritis (RA), a systemic disease of chronic inflammatory synovitis, leads to joint deformities, loss of function, and even death [1]. The pathogenesis of RA is yet unknown, but it is believed to be the result of the interaction of genetic, immune, and environmental factors [2]. Nonsteroidal anti-inflammatory drugs are the major clinical treatment for RA but can result in severe gastrointestinal reactions [3]. RA is a progressive process; therefore, drug treatments only slow its progression, but do not cure it [4]. Accordingly, searching for novel treatment strategies will be conducive to the treatment of RA patients.

MicroRNA (miRNA) is an evolutionarily conserved noncoding small RNA molecule, performing as a post-transcriptional gene regulator [5]. A single miRNA molecule is available to target hundreds of messenger RNAs (mRNAs) and participates in multiple physiological processes in the body [6]. Furthermore, dysregulated miRNAs have been the focus of RA research in recent years [7–9]. For instance, Fang et al. maintain that miR-92a suppresses proliferation and migration of fibroblast-like synovial cells in RA [10]. Hao et al. clarify that miR-135b-5p regulates inflammation in fibroblast-like synovial cells in RA [11]. Luo et al. [12] indicate that elevated miR-31 mediates proliferation and concentrations of interleukin (IL)-1β and tumor necrosis factor (TNF)-α in synovial cells. Proinflammatory cytokines are considerable in the progression of RA, as proinflammatory cytokines contribute to the onset of arthritis by promoting proteolytic enzyme activity that destroys the extracellular matrix of cartilage [13]. Suppressing the activity of proinflammatory cytokines has been a diagnostic index for lowering the incidence of arthritis [13, 14]. Distinctive miRNAs can suppress the release of proinflammatory factors, such as transforming growth factor (TGF)-β1, TNF-α, TGF-β, IL-8, and IL-6 [15–17]. Meanwhile, miR-345-3p can reduce expressions of cytokines (TNF-α and IL-6) to suppress inflammatory responses [18]. Nevertheless, the function of miR-345-3p on proinflammatory cytokines in patients with RA is yet unknown.

This study was to identify miRNAs impacting proinflammatory cytokines in RA, and miR-345-3p was a potential candidate. Moreover, the effect of miR-345-3p on HFLS-RA cell inflammation, proliferation, and apoptosis was evaluated, hoping to provide a reliable theoretical basis for miR-345-3p to improve the inflammatory response of RA.

## Materials and methods

### Subjects

From September 2018 to May 2019, 32 RA patients (RA group) including 15 males and 17 females who were admitted to Jiu Quan People’s Hospital were selected. The average age was (51.09 ± 11.07) years old, and the disease course was (10.06 ± 10.43) years. These RA patients met the RA classification criteria issued by the American College of Rheumatology in 2010 [5] and were not treated with glucocorticoids, immunosuppressants, and biological agents in the last six months. Patients with psychiatric disorders, tumors, severe organ damage, and severe RA were excluded, as were those who were pregnant, giving birth, or nursing. Another 32 healthy controls (HFLS group) from physical examination centers matched with age and sex were selected, all of which had no infection, immunity, and other possible RA-related diseases, including 16 males and 16 females, with an average age of (50.54 ± 10.03) years. From each subject, 5 mL peripheral blood was collected and centrifuged to obtain serums.

HFLS group had no history of systemic inflammation or tumor. The clinical data of all subjects were recorded, including age, gender, body mass index (BMI), erythrocyte sedimentation rate (ESR), serum uric acid (SUA), visual analogue scale (VAS), serum creatinine (SCR), leukocyte count, neutrophil count, and lymphocyte count. The protocol was authenticated by the Ethics Committee of Jiu Quan People's Hospital, and the written informed consent of each subject was obtained.

### RA clinical parameters

C-reactive protein (CRP), rheumatoid factor (RF), disease activity score 28 (DAS28), anti-cyclic citrullinated peptide (anti-CCP), leukocyte count, neutrophils count, tender joint count (TJC), and swollen joint count (SJC) were evaluated. Joint physical examinations of all patients were performed by doctors during outpatient visits.

ESR was determined using the Westfield method (mm/h), and CRP was tested by the automatic immunoturbidimetric method (mg/L). Anti-citrullinated protein antibody (ACPA) and RF were measured by Enzyme-linked immunosorbent assay (ELISA) kits (ThermoFisher Scientific, Waltham, Ma, USA). Other inspections were carried out on an automatic biochemical analyzer. Disease activity scores below 3.2 indicate low disease activity, while 3.2–5.1 and above 5.1 indicate moderate and high disease activity, separately.

### ELISA

Cytokines (TNF-α, TGF-β1, IL-6, IL-8) in patient’s serum were tested using ELISA kits (Thermo Fisher Scientific, USA). Serums were dropped into each well of the microtitration plate to determine the concentration of these molecules. Measurement of optical density (OD) at 450 nm was done on a microplate reader, with the wavelength corrected at 540 nm. Signals were detected with WHY101 microplate reader (power and Medical Systems Co.). Cytokine concentrations (pg/mL) were calculated in line with the standard curve [19].

### Reverse transcription quantitative polymerase chain reaction (RT-qPCR)

miR-345-3p and cytokines (TNF-α, TGF-β1, IL-6, IL-8) in patient’s serums, HFLS, and HFLS-RA cells were tested. Trizol reagent (Invitrogen, USA) was utilized to extract total RNA, and Prime Script RT kit (TaKaRaBIO) was purchased to perform reverse transcription with 1 μg total RNA. PCR was performed using real-time PCR Master Mix Kit (TOYOBO, Japan). Gene primers were shown in Table 1. U6 was an internal reference for miR-345-3p, and β-actin was an internal reference for mRNA [20].

**Table 1:** Genotyping primer sequences of polymerase chain reaction of miR-345-3p and correlated proinflammatory factors

Genes	Primer sequences (5′–3′)
MiR-345-3p	Forward: 5′-GGTTTTTGGATTGGGTTGTAGAGTG-3′
	Reverse: 5′-AACCAAAACAATCCCTTACCACTAC-3′
TGF-β1	Forward: 5′-CTTCAGCTCCACAGAGAAGAACTGC-3′
	Reverse: 5′-CACGATCATGTTGGACAACTGCTCC-3′
TNF-α	Forward: 5′-ACTGGCGTGTTCATCCGTTCT-3′
	Reverse: 5′-ACTGGCGTGTTCATCCGTTCT-3′
IL-8	Forward: 5′-GTCTGCTAGCCAGGATCCAC-3′
	Reverse: 5′-ACACAGCTGGCAATGACAAG-3′
IL-6	Forward: 5′-ACACAGCTGGCAATGACAAG-3′
	Reverse: 5′-TAGCCACTCCTTCTGTGACTCTAACT-3′
U6	Forward: 5′-CTCGCTTCGGCAGCACA-3′
	Reverse: 5′-AACGCTTCACGAATTTGCGT-3′
β-actin	Forward: 5′-TCCCATCACCATCTTCCA-3′
	Reverse: 5′-CATCACGCCACAGTTTTCC-3′

### Cell culture and transfection

HFLS-RA and HFLS were commercially gained (Jennio Biotechnology Co., Ltd.) and cultured in high-glucose-Dulbecco’s modified Eagle’s medium (ThermoFisher Scientific) or minimum essential medium (ThermoFisher Scientific) [21]. Transfection of the cells in the logarithmic growth phase was performed, and cells were seeded in 6-well plates with 5 × 10^5^ cells per well. Cells were divided into 6 groups: HFLS (PBS treatment), HFLS-RA, in-NC (HFLS-RA cells transfected with inhibitor-NC), miR-345-3p inhibitor (HFLS-RA cells transfected with miR-345-3p inhibitor), mir-NC (HFLS-RA cells transfected with mimic NC), and miR-345-3p mimic (HFLS-RA cells transfected with miR-345-3p mimic). Transfection was carried out in line with the instructions of lipofectamine 2000 kit (Invitrogen, USA). After 48 h, cells were harvested for follow-up experiments [22].

### Cell counting kit (CCK)-8 assay

CCK-8 kit (Dojindo, Kumamoto, Japan) tested HFLS and HFLS-RA cell proliferation. Cells were seeded into 96-well plates, mixed with CCK-8 reagent to each well at 0, 24, 48 and 72 h, and then incubated for 3 h. Absorbance was measured at 450 nm with a microplate reader (BioTek, Winooski, VT, USA) [23].

### Flow cytometry

Annexinv-fluorescein isothiocyanate (FITC)/PI double staining tested cell apoptosis. Cells were re-suspended in 200 μL binding buffer (BDBiosciences), mixed with 10 μL AnnexinV-FITC protein and 5 μL protein PI for 15 min, and added with 300 μL protein binding buffer. Apoptosis analysis was done using CoulterEpicsXL flow cytometer (Beckman Coulter, Chaska) at 488 nm wavelength [21].

### Statistical analysis

SPSS20.0 was used for data analysis, and GraphPadPrism6 was used for data visualization. Prism software was used to map the correlation between miR-345-3p expression and proinflammatory factors in RA patients, and the ROC curve of the diagnostic value of miR-345-3p and proinflammatory factors for RA. The area under the ROC curve (AUC) was calculated, and the sensitivity and specificity were obtained at the optimum cutoff value. The prognostic value of miR-345-3p levels and proinflammatory factors in RA patients was evaluated by Cox logistic regression analysis. Measurement data were expressed as mean ± SD. The measurement data conforming to normal distribution were compared between the two groups using independent sample t test. One-way analysis of variance and LSD-t test were for multiple group comparison. Multiple time points were compared by repeated measure analysis of variance. *P* < 0.05 was accepted as indicative of distinct differences.

## Results

### Baseline features and clinical parameters of participants

Table 2 shows the major features and laboratory results of the participants. A total of 64 participants, ranging in age from 41 to 63 years, were enrolled. No distinct differences were indicated in age, gender distribution, BMI, ESR, and lymphocyte count (*P* > 0.05). SUA, CRP, RF, leukocyte count, and neutrophil count were statistically different (*P* < 0.001). DAS28 between 3.2 and 5.1 indicated that the disease activity was moderate. The mean VAS of RA patients was 6.16 ± 2.36.

**Table 2:** Baseline features and clinical parameters of participants

Feature	HFLS (*n* = 32)	RA (*n* = 32)	*P*
Age (years)	50.54 ± 10.03	51.09 ± 11.07	0.850
Sex (male/female)	16/16	15/17	0.735
BMI (kg/m^2^)	20.72 ± 1.73	22.95 ± 1.64	0.553
ESR (mm/h)	4.68 ± 2.81	4.99 ± 3.01	0.517
SUA (umol/L)	185.15 ± 8.20	227.44 ± 12.36*	< 0.001
VAS (umol/L)	–	6.16 ± 2.36	–
SCR (umol/L)	89.34 ± 26.48	87.32 ± 26.27	0.600
Leukocyte count (109/L)	7.06 ± 1.83	25.92 ± 8.73*	< 0.001
Neutrophils count (103/mL)	4.49 ± 1.44	31.19 ± 9.05*	< 0.001
Lymphocytes count (109/L)	2.55 ± 0.94	2.29 ± 0.86	0.125
CRP (mg/L)	1.9 ± 0.9	19.48 ± 12.79*	< 0.001
RF (IU/mL)	5.02 ± 2.42	171.56 ± 145.8*	< 0.001
Anti-CCP (U/mL)	11.1 ± 5.1	273.6 ± 112.2*	< 0.001
DAS28	–	3.75 ± 0.94	–

*RA* Rheumatoid arthritis, *BMI* Body mass index, *ESR* Erythrocyte sedimentation rate, *SUA* Serum uric acid, *VAS* visual analogue scale, *SCR* serum creatinine, *CRP* C-reactive protein, *DAS28* Disease activity score, *RF* Rheumatoid factor, *Anti-CCP* Antioxidant citrullinate peptide, *SJC* Swollen joint count, *TJC* Tender joint count; Data were expressed as mean ± SD. * *P* < 0.05 compared with HFLS group

### miR-345-3p is lowly expressed in RA and negatively correlated with VAS

**Fig. 1:**
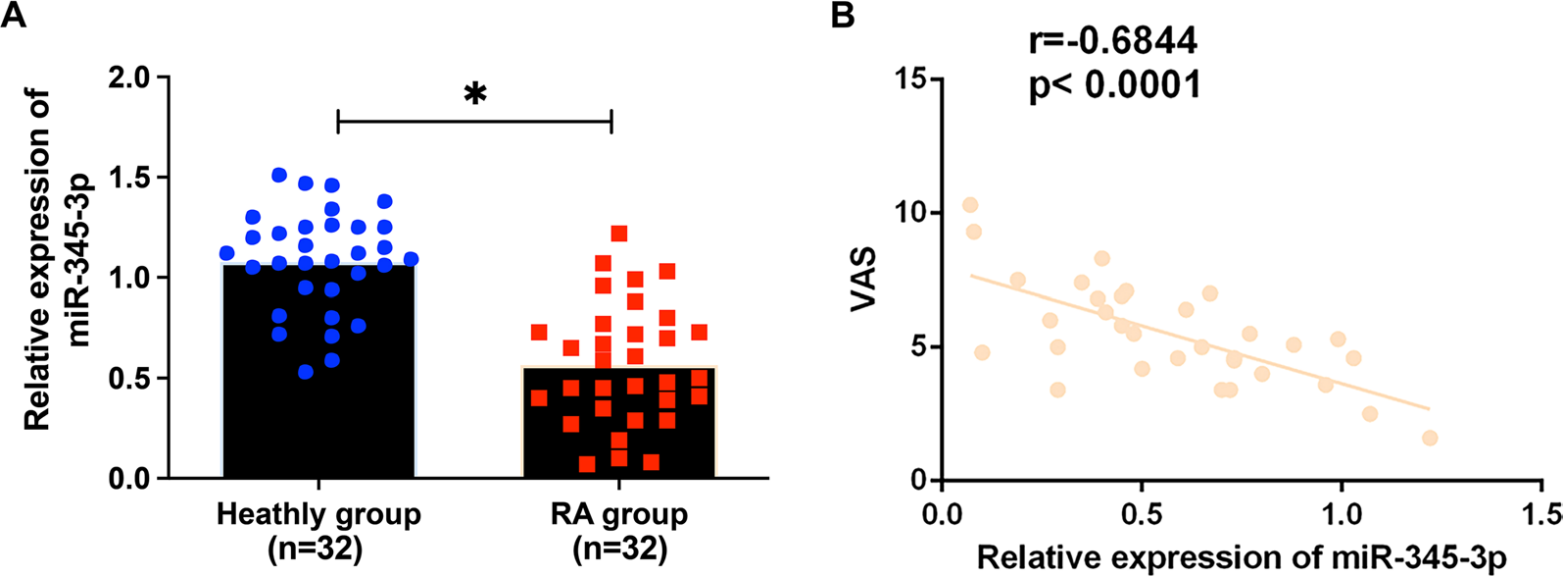
MiR-345-3p is in RA patients and its correlation with VAS. RT-qPCR measured miR-345-3p in the serum of RA patients and healthy controls (**A**); **B** Negative correlation between miR-345-3p and VAS in RA patients (*r* = − 0.7671, *P* < 0.0001); Data were expressed as mean ± SD. *P < 0.05

MiR-345-3p in the serum of RA patients was lower than in healthy controls (Fig. 1A, *P* < 0.05). Furthermore, miR-345-3p was supposed to be a critical biomolecule of RA. To further explore the association between miR-345-3p with RA, the correlation between VAS and miR-345-3p was also studied. Serum miR-345-3p in RA patients was negatively correlated with VAS (*r* = − 0.6844, *P* < 0.0001), as presented in Fig. 1B. In brief, miR-345-3p was supposed to be associated with the occurrence and severity of RA.

### miR-345-3p is negatively correlated with inflammatory cytokines in RA patients

**Fig. 2:**
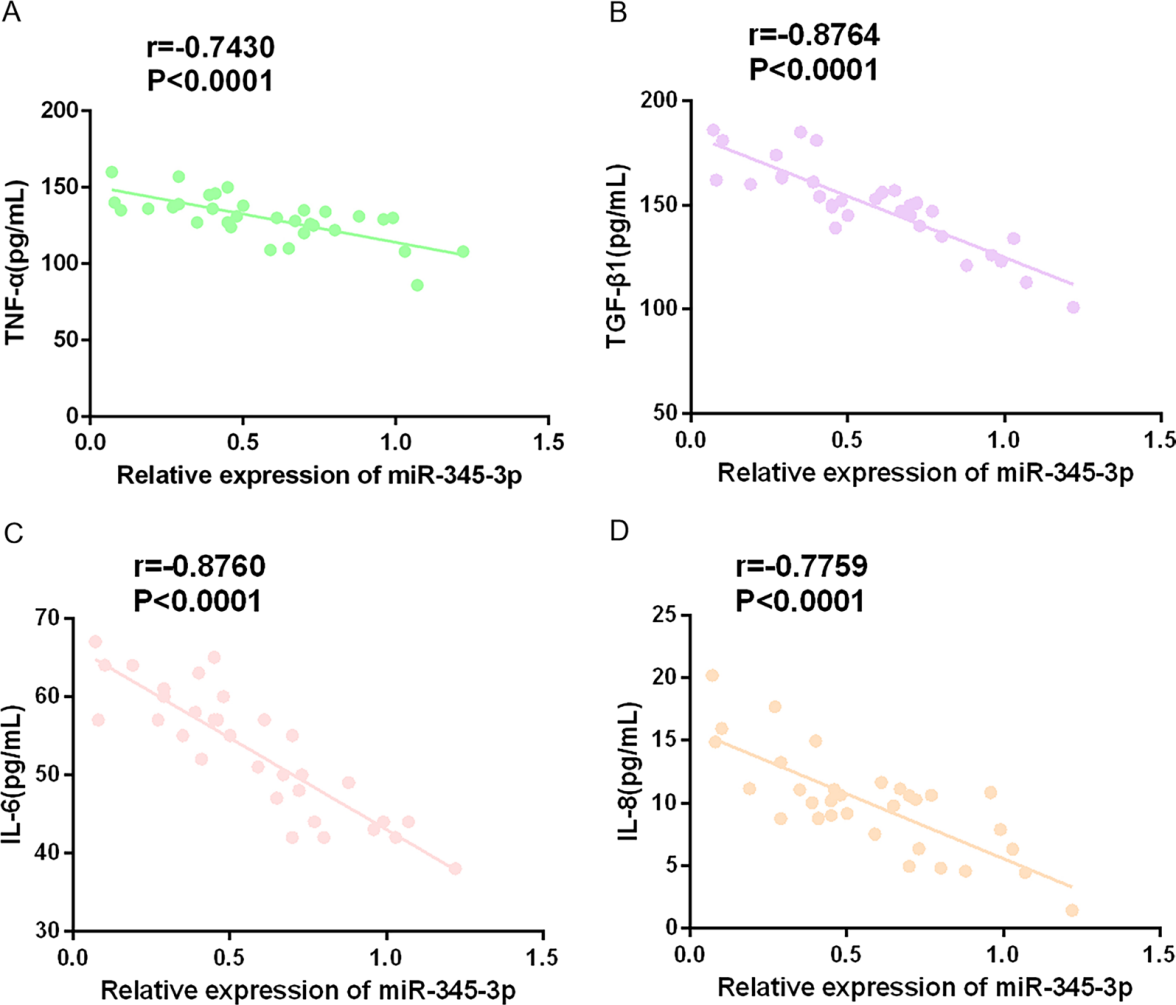
Correlation is of miR-345-3p with IL-8, TGF-β1, TNF-α and IL-6. Pearson correlation analysis of miR-345-3p with TGF-β1 (**A**), TNF-α (**B**), IL-8 (**C**), and IL-6 (**D**)

Proinflammatory factors are involved in the pathogenesis of RA [24–28]. The association between miR-345-3p and inflammatory factors in patients with RA was evaluated by Pearson correlation analysis. The results showed that miR-345-3p and TNF-α (Fig. 2A, r = − 0.7430, *P* < 0.0001), TGF-β1 (Fig. 2B, *r* = − 0.8764, *P* < 0.0001), IL-6 (Fig. 2C, *r* = − 0.8760, *P* < 0.0001), and IL-8 (Fig. 2D, *r* = − 0.7759, *P* < 0.0001) were negatively correlated. These data indicate that miR-345-3p is highly correlated with the level of inflammatory cytokines in RA patients, and it may be involved in regulating the release of inflammatory cytokines (Table 3).

**Table 3:** ELISA results of proinflammatory factors in RA patients

Variable	Expression (mean ± SD) (pg/mL)
TNF-α	129.97 ± 11.74
TGF-β1	150.16 ± 19.24
IL-6	53.06 ± 9.43
IL-8	10.00 ± 1.87

### miR-345-3p and proinflammatory factors have high diagnostic value in RA

**Fig. 3:**
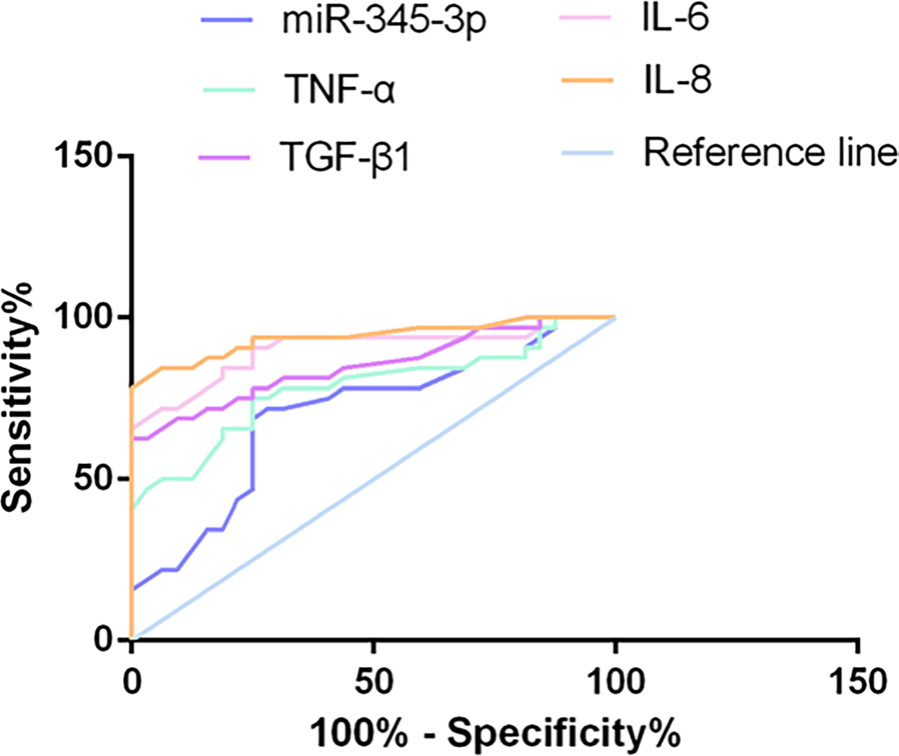
Diagnostic value of miR-345-3p and proinflammatory factors in RA patients. ROC curve analysis of miR-345-3p, TGF-β1, TNF-α, IL-6, and IL-8 in RA patients and healthy controls

ROC curve was drawn based on miR-345-3p expression in RA patients and healthy controls to assess the diagnostic value of miR-345-3p. As proved in Fig. 3 and Table 4, miR-345-3p was available to be adopted to distinguish RA patients from healthy people (AUC = 0.7012). Additionally, TGF-β1 (AUC = 0.8506), TNF-α (AUC = 0.7827), IL-6 (AUC = 0.9395), and IL-8 (AUC = 0.9019) were also provided with diagnostic values for RA patients.

**Table 4:** ROC curve analysis results of miR-345-3p and proinflammatory factors in RA patients

Variable	AUC	Sensitivity (%)	Specificity (%)
MiR-345-3p	0.7012	71.88	71.88
TNF-α	0.7827	75.00	71.88
TGF-β1	0.8506	78.13	75.00
IL-6	0.9395	90.63	78.13
IL-8	0.9019	87.50	75.00

ESR and CRP, non-specific inflammatory markers, have been adopted to assess systemic inflammation and are provided with diagnostic values for RA [29]. In the early diagnosis of RA, the diagnostic accuracy was improved by detecting anti-CCP antibody and RF [30, 31]. DAS28 is a quantitative index for assessing RA disease activity, integrating information from joint swelling, tender joints, acute phase response, and general health [32]. TJC and SJC are effective tools for testing joint tenderness and swelling [33]. MiR-345-3p and the disease activity indices DAS-28, SJC, TJC, and clinical parameters such as CRP and ESR were analyzed by multivariate Cox analysis. CRP, ESR, anti-CCP, RF, DAS28, TJC, and SJC were predictors of RA patients, while miR-345-3p, TNF-α, TGF-β1, IL-6, and IL-8 were independent prognostic factors for the survival of RA patients (Table 5).

**Table 5:** Multivariate Cox regression analysis of RA patients

Feature	Multivariate analysis		
	HR	95% CI	*P*
MiR-345-3p	3.17	(1.82–5.12)	0.014
TNF-α (umol/L)	2.55	(0.88, 2.00)	0.041
TGF-β1(umol/L)	2.58	(1.20, 3.93)	0.045
IL-6 (umol/L)	2.82	(1.20, 2.50)	0.025
IL-8 (umol/L)	2.35	(1.10, 2.81)	0.041
CRP (mg/L)	1.02	(1.00, 1.04)	0.001
ESR (mm/h)	1.01	(1.00, 1.10)	0.0194
Anti-CCP (umol/L)	2.24	(1.32, 3.79)	0.047
RF (umol/L)	1.00	(1.003, 1.007)	< 0.0001
DAS28	1.40	(1.00, 1.90)	0.028
TJC	1.10	(1.00, 1.50)	0.022
SJC	1.12	(0.84, 1.21)	0.032

*HR* Hazard ratio, *TGF* Transforming growth factor, *TNF* Tumor necrosis factor, *IL* Interleukin

### miR-345-3p regulates the levels of inflammatory cytokines in HFLS-RA cells

**Fig. 4:**
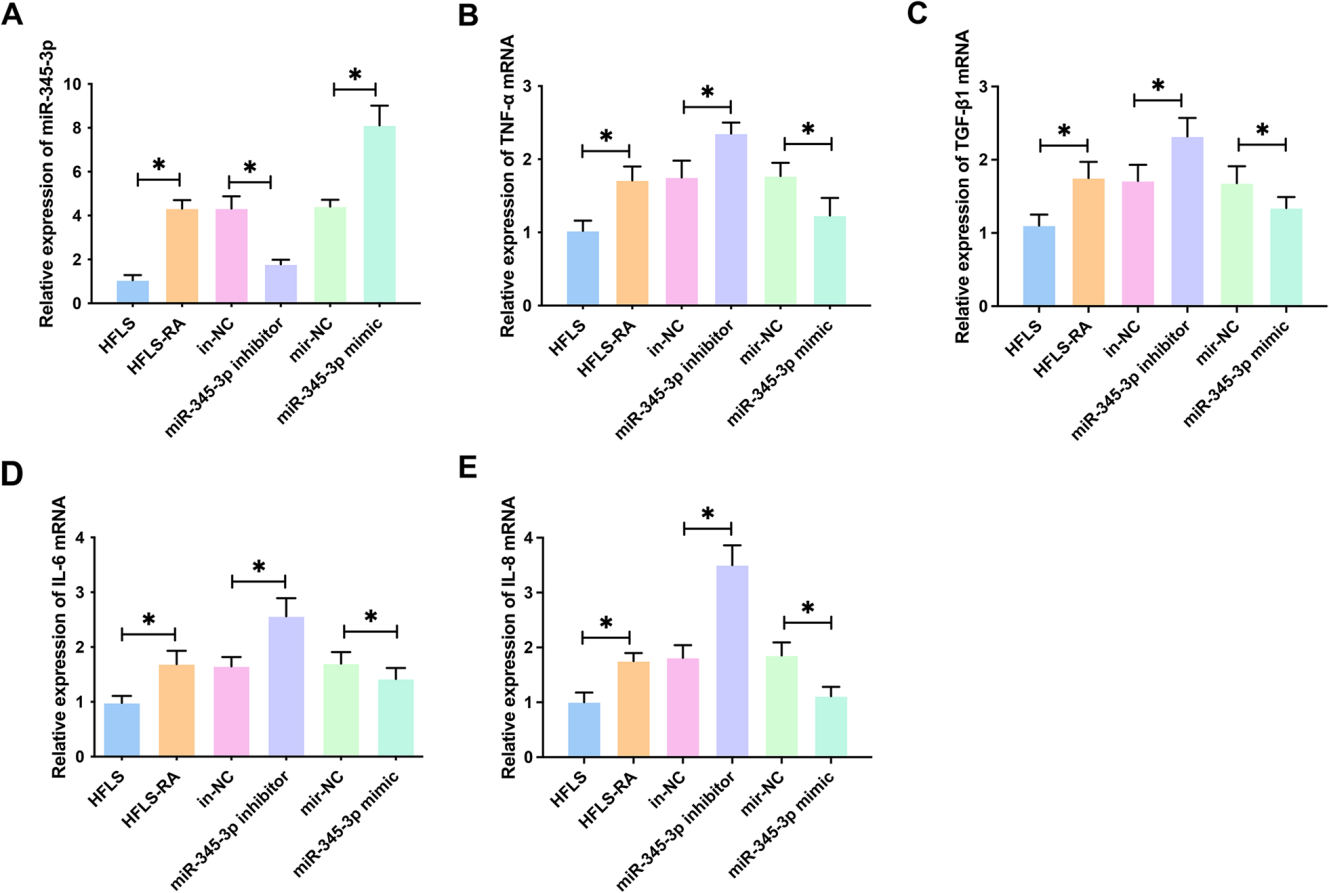
MiR-345-3p mediates proinflammatory factors in HFLS-RA cells. RT-qPCR measured miR-345-3p (**A**), TGF-β1 (**B**), TNF-α (**C**), IL-6 (**D**), and IL-8 (**E**); Data were expressed as mean ± SD. *P < 0.05

To explore the effect of miR-345-3p on proinflammatory factors in HFLS-RA cells, miR-345-3p in cells of each group was analyzed by RT-qPCR. miR-345-3p in HFLs-RA cells was higher than that in HFLS cells. Transfection of miR-345-3p mimic and miR-345-3p inhibitor promoted and inhibited miR-345-3p expression in HFLS-RA cells, respectively (Fig. 4A). Subsequently, mRNA expression of inflammatory cytokines was evaluated. TNF-α (Fig. 4B), TGF-β1 (Fig. 4C), IL-6 (Fig. 4D), and IL-8 (Fig. 4E) in HFLS-RA cells were higher than those in HFLS cells. Knockdown and overexpression of miR-345-3p suppressed and induced their expression trend, respectively (Fig. 4B–E). These data indicate that miR-345-3p can regulate the release of inflammatory cytokines in HFLS-RA cells.

### miR-345-3p influences HFLS-RA cell proliferation and apoptosis

**Fig. 5:**
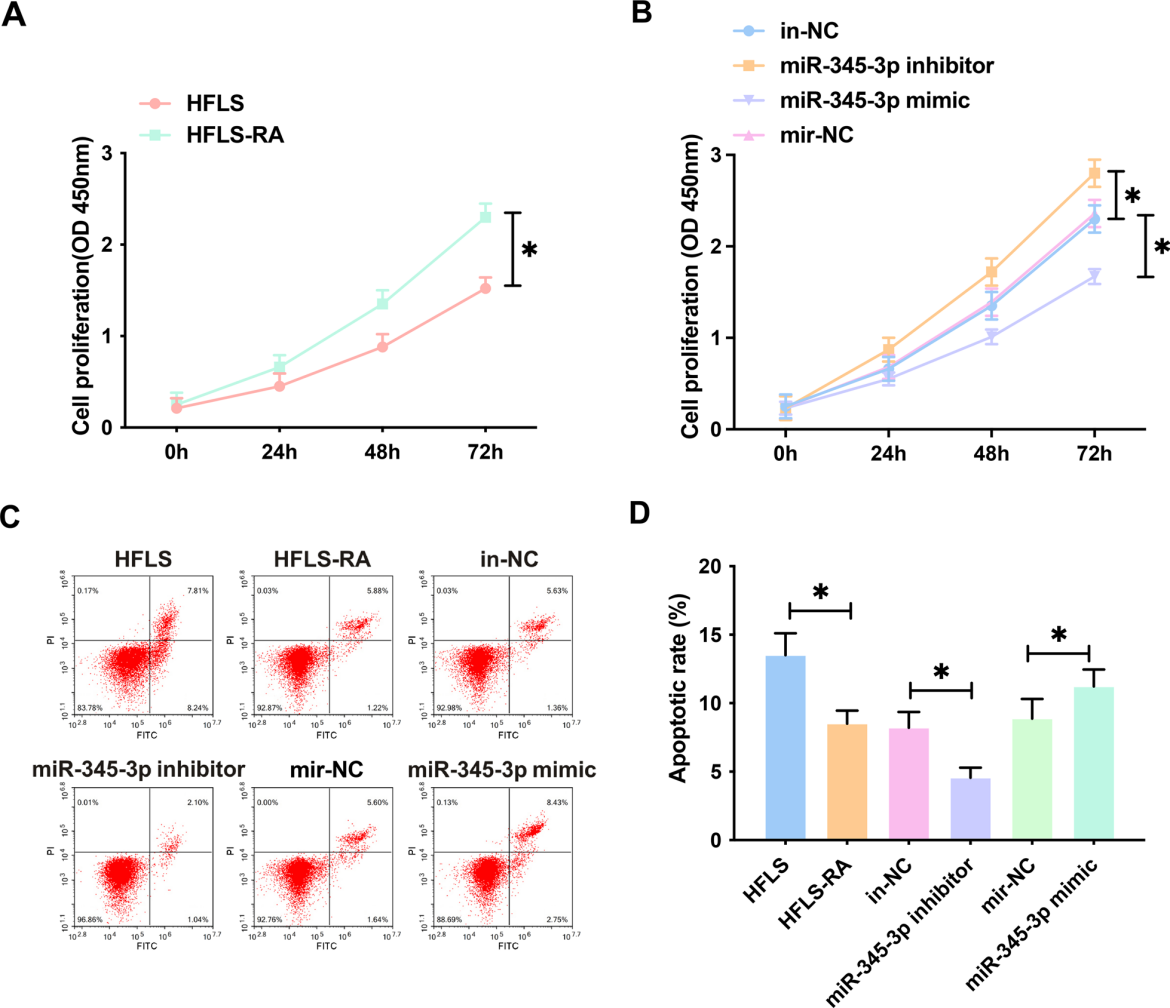
MiR-345-3p mediates proliferation and apoptosis in HFLS-RA cells. CCK-8 assayed proliferation capacity (**A**–**B**); Flow cytometry detected apoptosis (**C**); Data were expressed as mean ± SD. *P < 0.05

Proinflammatory factors are available to stimulate the proliferation and activation of fibroblast-like synaptic cells, destroy cartilage, and induce the destruction of joint structures [13]. Therefore, the effects of miR-345-3p on the proliferation and apoptosis of HFLS-RA were further evaluated. The proliferation rate of HFLs-Ra was significantly higher than that of HFLS (Fig. 5A). In addition, overexpression or knockdown of miR-345-3p inhibited and promoted the proliferation rate of HFLS-RA, respectively (Fig. 5B). Flow cytometry showed that overexpression of miR-345-3p increased the apoptosis rate of HFLS-RA, but knockdown of miR-345-3p had the opposite effect (Fig. 5C).

## Discussion

RA is a multi-organ inflammatory autoimmune disease influencing multiple organ systems [34, 35]. At present, the clinical therapeutic effect remains to be improved [36]. Accordingly, searching for new treatment strategies helps to ameliorate the quality of treatment for RA patients. In the past few years, multiple studies have clarified that miRNAs exert a critical part in various autoimmune diseases [37, 38]. Additionally, miRNAs are also available to modulate joint inflammation in RA. For instance, miR-23a-5p modulates inflammation of MH7A synaptic cells via targeting TLR4 in RA [39]. In short, miRNAs are supposed to have the potential as new biomarkers for RA treatment.

In this study, miR-345-3p as a potential biomarker for RA treatment was discovered. Although many serum markers of RA are used for surveillance and diagnosis, proper assessment of RA remains difficult, especially in the early stages of the disease, due to its inconspicuous sensitivity and non-specificity [40]. Studies have clarified that particular miRNAs, as biomarkers of RA, facilitate disease diagnosis and prognosis prediction, such as miR-223 [41], has-miR-1915-3p [42], miR-125a, and miR-125-b [43]. Additionally, proinflammatory factors have also been broadly adopted as biomarkers for the diagnosis and treatment of RA in the past few years [24, 26, 28, 44, 45]. ROC curve analysis and multivariate Cox analysis confirmed that miR-345-3p and proinflammatory factors had high diagnostic ability and predictive value in RA patients. Kaplan–Meier survival curve found that patients with high expression levels of miR-345-3p had better overall survival.

Primarily, miR-345-3p in the serum of RA patients was found to be low. Multiple studies have testified that VAS is a critical index for clinical assessment of RA [46–48]. Here, it was found that miR-345-3p was negatively correlated with VAS, suggesting that miR-345-3p may be related to the occurrence and severity of RA. Proinflammatory factors TNF-α, TGF-β1, IL-6, and IL-8 are involved in the pathogenesis of RA and are supposed to generate a protective function [24–28]. This study found that miR-345-3p was negatively correlated with proinflammatory cytokines TNF-α, TGF-β1, IL-6, and IL-8.

HFLS-RA (rheumatoid arthritis fibroblast synaptic cell) model was used for cell experiments to better verify the experimental results [21, 27, 49]. miR-345-3p expression was interfered with in HFLS-RA to evaluate the effects of miR-345-3p on proinflammatory factors and related biological functions. As studied, overexpressing miR-345-3p could down-regulate proinflammatory factors in HFLS-RA cells, promoting cell apoptosis and suppressing cell proliferation. Studies have mentioned that miRNAs mediate RA progression and associated inflammatory response through targeting mRNAs [50, 51]. Therefore, in the future, it is necessary to further explore the targeted regulatory pathway of miR-345-3p on the expression of proinflammatory factors.

In short, elevated miR-345-3p restrained proinflammatory cytokines in patients with RA, thereby exerting a protective function on RA diseases. Nevertheless, limitations still remained. Initially, the research population should be expanded to better testify the research results. Additionally, the partial role of miR-345-3p in HFLS-RA cells was only studied, and the particular mechanism of miR-345-3p in RA patients was not explored. Besides that, animal experiments were not conducted. Consequently, a superior complete experimental analysis will be carried out later to fix all the above limitations.
